# Predicting Managers' Mental Health Across Countries: Using Country-Level COVID-19 Statistics

**DOI:** 10.3389/fpubh.2022.791977

**Published:** 2022-05-19

**Authors:** Lun Li, Stephen X. Zhang, Lorenz Graf-Vlachy

**Affiliations:** ^1^School of Economics and Management, Tsinghua University, Beijing, China; ^2^Adelaide Business School, University of Adelaide, Adelaide, SA, Australia; ^3^TU Dortmund University, Dortmund, Germany; ^4^ESCP Business School, Paris, France

**Keywords:** managers, mental disorders, cumulative deaths, COVID-19, cross-country

## Abstract

**Background:**

There is limited research focusing on publicly available statistics on the Coronavirus disease 2019 (COVID-19) pandemic as predictors of mental health across countries. Managers are at risk of suffering from mental disorders during the pandemic because they face particular hardship.

**Objective:**

We aim to predict mental disorder (anxiety and depression) symptoms of managers across countries using country-level COVID-19 statistics.

**Methods:**

A two-wave online survey of 406 managers from 26 countries was performed in May and July 2020. We used logistic panel regression models for our main analyses and performed robustness checks using ordinary least squares regressions. In the sample, 26.5% of managers reached the cut-off levels for anxiety (General Anxiety Disorder-7; GAD-7) and 43.5% did so for depression (Patient Health Questionnaire-9; PHQ-9) symptoms.

**Findings:**

We found that cumulative COVID-19 statistics (e.g., cumulative cases, cumulative cases per million, cumulative deaths, and cumulative deaths per million) predicted managers' anxiety and depression symptoms positively, whereas daily COVID-19 statistics (daily new cases, smoothed daily new cases, daily new deaths, smoothed daily new deaths, daily new cases per million, and smoothed daily new cases per million) predicted anxiety and depression symptoms negatively. In addition, the reproduction rate was a positive predictor, while stringency of governmental lockdown measures was a negative predictor. Individually, we found that the cumulative count of deaths is the most suitable single predictor of both anxiety and depression symptoms.

**Conclusions:**

Cumulative COVID-19 statistics predicted managers' anxiety and depression symptoms positively, while non-cumulative daily COVID-19 statistics predicted anxiety and depression symptoms negatively. Cumulative count of deaths is the most suitable single predictor of both anxiety and depression symptoms. Reproduction rate was a positive predictor, while stringency of governmental lockdown measures was a negative predictor.

## Introduction

Since the outbreak of COVID-19, many studies have examined the pandemic's influence on the general public's mental health in various countries (1–6). This stream of research predominantly studied predictors of mental health at the individual level, for example, demographic characteristics (6, 7).

Further, scholars have recently begun to focus on the mental health of specific groups, most prominently healthcare workers (4, 8–11), but also students (12), hospitality workers (13), footballers (14), etc. However, this research again studies non-country-level predictors and, critically, there is hardly any research focused on the specific group of managers (15). This is problematic because managers perform one of the most stressful and consequential jobs (16, 17). For one, during a pandemic, managers cannot manage as usual, and they may thus suffer particularly due to the decision-making and leadership responsibilities they must exercise during such a time of crisis (18). For another, managers' mental health may also have important second-order effects on their subordinates' lives and therefore their subordinates' mental health (15).

In addition, the majority of research on mental health during COVID-19 uses cross-sectional data (2, 6, 19), and several systematic reviews across countries (20–23) have revealed that there exists no cross-country research studying the link between the severity of the pandemic and managers' mental health. It is thus novel and likely practically useful to track changes in managers' mental health during a pandemic and identify the most suitable predictors for it (24).

This research aims to use country-level COVID-19 statistics to predict managers' anxiety and depression symptoms using two-wave online survey data. It is among the first to focus on the group of vulnerable managers (15), and the first to do so using longitudinal data. We first examine the predictive capabilities of different country-level pandemic severity statistics and then compare their effects on model fit to identify the most suitable predictor of mental health issues.

## Methods

### Sample and Procedure

We implemented a two-wave online survey to collect data from managers. The first-wave survey was conducted on May 2, 2020, and the second-wave survey was conducted on June 17, 2020. Respondents to our survey are former consultants of a global management consulting firm who moved into managerial roles after consulting. After dropping observations that had missing data in our country-level predictors, we had a total of 812 usable responses from 406 managers. Mean levels of anxiety and depression symptoms varied substantially across countries and over time in our sample. For example, the prevalence of anxiety and depression symptoms for the US was 13 and 14%, respectively, in the first-wave survey, and 52 and 79%, respectively, in the second-wave survey. In contrast, the prevalence of anxiety and depression for Japan was 0% in the first-wave survey; and the prevalence of anxiety and depression for Japan was 20 and 80% in the second-wave survey, respectively. Such increases were presumably driven by the constant pressures the pandemic exerted on managers over time. All managers participated voluntarily in the survey, which they could terminate at any time, and were not compensated. The study was approved by the responsible body at ESCP Business School (#2020-04-01 and #2020-06-01).

### Measures for Individual-Level Variables

We measured anxiety using the generalized anxiety disorder (GAD-7) instrument, which consists of seven questions (α = 0.88), with a cutoff of 10 or greater indicating anxiety disorder symptoms (25). Depression was assessed using the Patient Health Questionnaire (PHQ-9), which consists of nine questions (α = 0.83), with a cutoff of 10 or greater indicating depression disorder symptoms (26, 27).

We also collected socio-demographic information (28), including gender (categorical), age in years (continuous), educational level (ordinal: completed less than secondary school/completed secondary school/attended some college but no degree/completed college degree/completed graduate degree), and number of children (continuous).

### Measures for Country-Level Variables

We obtained cumulative counts of confirmed cases, cumulative counts of confirmed cases per million, cumulative counts of deaths, cumulative counts of deaths per million, daily counts of new confirmed cases, daily counts of new deaths, daily counts of new confirmed cases per million, and daily counts of new deaths per million for each country from the Coronavirus Resource Center (CRC) at Johns Hopkins University. All data was obtained for the exact day each manager responded to the survey.

We calculated smoothed values of daily counts of new confirmed cases, daily counts of new deaths, daily counts of new confirmed cases per million, and daily counts of new deaths per million by taking the mean of the daily values in the week prior to managers' responses. All absolute cumulative counts and daily new counts were log-transformed to account for the skewed distribution of the data. We obtained the reproduction rate of COVID-19 (i.e., the estimated number of new infections caused by a single infected individual) and a stringency index that measures the strictness of governments' lockdown policies for each country from Roser et al. (29). This stringency index comprises nine indicators, including school closures, workplace closures, and travel bans, and ranges from 0 (no policies) to 100 (very strict policies). To control for potential general country effects on mental health, we also considered population density and gross domestic product (GDP) per capita and obtained corresponding data from the World Bank.

### Analysis

As our data on individuals' mental health is nested in the two-wave survey across 26 countries, we employed panel analysis (30). Specifically, we ran panel logistic regression analyses with dummy variables for countries using Stata 17 to predict managers' mental health issues at a significance level of 0.05.

We ran models separately for anxiety and depression. Specifically, for each dependent variable, we ran a model that included all individual-level predictors, and then 14 models that included all individual-level predictors and one country-level predictor each. We used AIC and BIC to identify the models with the best fit, and thus identify the most suitable country-level predictors for anxiety and depression, respectively.

## Results

### Descriptive Findings

Tables 1, 2 present descriptive statistics of the sampled managers and COVID-19 severity statistics across different countries. The mean scores of anxiety (GAD-7) and depression (PHQ-9) were 0.26 (SD = 0.44) and 0.43 (SD = 0.50), respectively. Overall, the proportion of our sampled participants with anxiety disorder symptoms is much lower (*p* = 0.000) in the first-wave survey (7.6%) compared to the second-wave survey (45.3%). Similarly, the prevalence of depression disorder symptoms is much lower (*p* = 0.000) in the first-wave survey (8.1%) compared to the second-wave survey (78.8%).

**Table 1:** Descriptive statistics (*n* = 812, *N* = 406).

**Variable**	**Frequency (%)**
**Anxiety symptoms**
No	597 (73.5%)
Yes	215 (26.5%)
**Depression symptoms**
No	459 (56.5%)
Yes	353 (43.5%)
**Gender**
Male	306 (75.4%)
Female	100 (24.6%)
**Age**
25–34	28 (6.9%)
35–44	142 (35.0%)
45–54	150 (37.0%)
55–64	57 (14.0%)
65 or above	29 (7.1%)
**Education**
Bachelor's degree	30 (7.4%)
Master's degree	376 (92.6%)
**Children**
0	163 (40.2%)
1	65 (16.0%)
2	130 (32.0%)
3	41 (10.1%)
4	7 (1.7%)
**Country**
United States	230 (28.3%)
Germany	152 (18.7%)
Switzerland	52 (6.4%)
Australia	50 (6.2%)
United Kingdom	48 (5.9%)
France	40 (4.9%)
India	26 (3.2%)
Italy	26 (3.2%)
Spain	24 (2.5%)
Japan	20 (2.2%)
Belgium	18 (2.2%)
Netherlands	18 (2.2%)
Singapore	18 (2.2%)
Sweden	18 (2.2%)
Canada	16 (2.0%)
Portugal	12 (1.5%)
Austria	10 (1.2%)
Others	34 (4.2%)

*n indicates the number of observations, N indicates the number of managers*.

**Table 2:** Descriptives for mental disorder symptoms and country-level COVID-19 statistics.

	**Full sample**		**United States**		**Germany**		**Switzerland**		**Australia**		**United Kingdom**		**France**		**India**		**Italy**	
**Variable**	**Mean**	**SD**	**Mean**	**SD**	**Mean**	**SD**	**Mean**	**SD**	**Mean**	**SD**	**Mean**	**SD**	**Mean**	**SD**	**Mean**	**SD**	**Mean**	**SD**
Anxiety (GAD-7)	0.265	0.441	0.326	0.470	0.243	0.431	0.173	0.382	0.180	0.388	0.250	0.438	0.225	0.423	0.231	0.430	0.154	0.368
Depression (PHQ-9)	0.435	0.496	0.465	0.500	0.408	0.493	0.346	0.480	0.360	0.485	0.417	0.498	0.400	0.496	0.423	0.504	0.385	0.496
Population density	325.869	1,150.883	35.608	0.000	237.016	0.000	214.243	0.000	3.202	0.000	272.898	0.000	122.578	0.000	450.419	0.000	205.859	0.000
GDP per capita	45,387.553	12,905.984	54,225.446	0.000	45,229.245	0.000	57,410.166	0.000	44,648.710	0.000	39,753.244	0.000	38,605.671	0.000	6,426.674	0.000	35,220.084	0.000
Cumulative cases (log)	12.004	1.753	14.218	0.267	12.064	0.062	10.320	0.018	8.858	0.034	12.310	0.184	11.866	0.081	11.589	0.943	12.309	0.060
Cumulative cases per million	3,013.093	1,752.177	4,681.441	1,223.992	2,073.820	127.591	3,505.638	64.347	275.684	9.385	3,322.599	602.455	2,187.716	176.493	114.138	85.648	3,672.525	221.180
Cumulative deaths (log)	9.082	2.192	11.367	0.251	8.943	0.129	7.354	0.064	4.593	0.043	10.409	0.161	10.204	0.080	8.102	0.863	10.346	0.088
Cumulative deaths per million	228.155	185.889	269.437	66.225	92.103	11.744	180.735	11.469	3.838	0.167	494.563	78.590	415.088	32.976	3.301	2.345	516.958	45.207
Daily new cases (log)	6.998	2.463	10.125	0.171	6.312	0.452	3.570	0.987	2.474	0.498	7.575	0.774	6.429	0.421	8.620	0.641	6.638	0.930
Smoothed daily new cases (log)	7.058	2.414	10.108	0.137	6.463	0.591	3.819	1.030	2.442	0.229	7.718	0.689	6.445	0.461	8.429	0.772	6.652	0.961
Daily new deaths (log)	4.293	2.390	6.995	0.441	3.902	0.779	1.362	1.369	0.155	0.321	5.390	1.085	4.482	0.991	5.245	0.627	5.059	0.816
Smoothed daily new deaths (log)	4.487	2.313	7.146	0.397	4.071	0.873	1.737	1.242	0.487	0.497	5.707	0.715	4.816	0.901	4.991	0.650	5.044	0.755
Daily new cases per million	33.694	34.793	76.492	13.000	7.222	3.002	6.093	4.977	0.481	0.234	37.569	25.232	10.225	3.708	4.816	2.683	17.962	12.906
Smoothed daily new cases per million	33.944	32.720	74.829	10.279	9.013	4.854	8.146	6.495	0.423	0.097	41.077	24.566	10.606	4.400	4.303	2.790	18.910	14.251
Daily new deaths per million	2.209	2.368	3.613	1.541	0.765	0.567	0.949	1.346	0.009	0.020	5.054	3.908	2.051	1.716	0.161	0.083	3.533	2.730
Smoothed daily new deaths per million	2.513	2.468	4.134	1.561	0.970	0.694	1.095	1.027	0.033	0.034	5.562	3.407	2.671	1.941	0.128	0.074	3.285	2.122
Reproduction rate	0.903	0.185	0.976	0.044	0.784	0.114	0.757	0.223	0.889	0.222	0.802	0.094	1.032	0.047	1.315	0.100	0.720	0.053
Stringency index	69.664	11.241	72.658	0.345	66.202	7.105	59.186	10.374	64.165	6.332	75.850	4.096	80.090	7.970	83.047	8.515	67.271	23.430
	**Spain**		**Japan**		**Belgium**		**Netherlands**		**Singapore**		**Sweden**		**Canada**		**Portugal**		**Austria**		**Others**	
**Variable**	**Mean**	**SD**	**Mean**	**SD**	**Mean**	**SD**	**Mean**	**SD**	**Mean**	**SD**	**Mean**	**SD**	**Mean**	**SD**	**Mean**	**SD**	**Mean**	**SD**	**Mean**	**SD**
Anxiety (GAD-7)	0.292	0.464	0.100	0.308	0.333	0.485	0.444	0.511	0.222	0.428	0.167	0.383	0.125	0.342	0.333	0.492	0.400	0.516	0.412	0.500
Depression (PHQ-9)	0.417	0.504	0.400	0.503	0.611	0.502	0.444	0.511	0.556	0.511	0.444	0.511	0.375	0.500	0.417	0.515	0.500	0.527	0.588	0.500
Population density	93.105	0.000	347.778	0.000	375.564	0.000	508.544	0.000	7,915.731	0.000	24.718	0.000	4.037	0.000	112.371	0.000	106.749	0.000	103.260	100.454
GDP per capita	34,272.360	0.000	39,002.223	0.000	42,658.576	0.000	48,472.545	0.000	85,535.383	0.000	46,949.283	0.000	44,017.591	0.000	27,936.896	0.000	45,436.686	0.000	18,631.652	9,351.839
Cumulative cases (log)	12.348	0.050	9.688	0.070	10.922	0.081	10.689	0.083	10.184	0.388	10.390	0.366	11.234	0.243	10.304	0.168	9.690	0.043	9.624	1.760
Cumulative cases per million	4,934.179	247.267	127.761	8.937	4,792.748	388.819	2,569.374	213.236	4,850.247	1,794.763	3,429.115	1,216.803	2,059.805	488.935	2,964.486	495.202	1,795.634	76.425	710.180	797.705
Cumulative deaths (log)	10.177	0.034	6.530	0.302	9.077	0.084	8.614	0.092	3.077	0.186	8.274	0.239	8.609	0.368	7.125	0.188	6.454	0.061	6.356	1.653
Cumulative deaths per million	562.475	18.989	5.648	1.657	757.455	63.333	322.536	29.587	3.599	0.687	398.621	93.255	154.504	54.100	123.798	22.948	70.550	4.295	25.804	29.335
Daily new cases (log)	6.188	0.728	4.559	0.865	5.311	0.572	5.527	0.418	6.191	0.439	6.518	0.560	6.811	0.507	5.206	1.667	3.500	0.735	5.805	2.243
Smoothed daily new cases (log)	6.368	0.705	4.600	0.885	5.435	0.695	5.559	0.478	6.266	0.264	6.542	0.254	6.961	0.482	5.614	0.168	3.643	0.249	5.709	2.229
Daily new deaths (log)	2.751	2.703	1.850	1.342	3.491	0.996	3.100	1.317	0.231	0.336	3.978	0.391	4.626	0.678	2.015	1.203	1.160	0.747	2.715	2.004
Smoothed daily new deaths (log)	3.190	2.500	2.200	0.932	3.753	0.969	3.351	1.118	0.320	0.173	3.991	0.390	4.785	0.368	2.584	0.412	1.301	0.903	2.720	1.915
Daily new cases per million	13.189	8.685	1.046	0.814	20.703	14.692	15.942	7.115	91.001	38.874	77.030	40.262	26.982	12.853	27.305	11.336	4.475	2.847	20.517	24.614
Smoothed daily new cases per million	15.581	9.810	1.090	0.786	24.376	14.797	16.753	7.570	92.780	23.639	70.738	18.162	31.027	13.931	27.121	4.317	4.249	1.079	17.458	20.628
Daily new deaths per million	2.379	2.529	0.093	0.103	4.189	3.339	2.474	2.428	0.057	0.083	5.572	2.106	3.241	1.824	1.185	1.361	0.333	0.296	0.770	1.039
Smoothed daily new deaths per million	3.018	3.120	0.094	0.074	5.315	4.048	2.685	2.242	0.068	0.042	5.648	2.133	3.339	1.131	1.298	0.509	0.458	0.429	0.772	1.203
Reproduction rate	0.812	0.061	0.818	0.245	0.737	0.038	0.815	0.169	0.898	0.089	1.104	0.034	0.867	0.106	0.983	0.099	0.814	0.209	1.165	0.230
Stringency index	71.124	12.732	37.960	9.501	73.456	10.591	71.295	8.577	81.485	3.812	60.493	1.795	71.760	0.960	75.695	7.708	56.015	9.271	73.845	16.215

Of the 406 managers, 75.4% (306) were male. All held at least bachelor's degrees. Age ranged from 29 to 78 years. Most managers (40.2%) had no children.

For about half of the participants (44.6%), their countries' population density ranged between 1 and 100. GDP per capita in the countries of most managers (69.5%) ranged from 40,000 to 60,000 (constant 2011 US dollars). The reproduction rate of the COVID-19 epidemic ranged from 0.51 to 1.50, and the stringency index ranged from 28.7 to 96.3 across the 26 countries over the survey waves.

### Predictors of Managers' Mental Health

As presented in Tables 3, 4, female managers were more likely than male managers to exhibit anxiety and depression symptoms. Age negatively predicted managers' anxiety and depression. The effects of education level and the number of children were not significant.

**Table 3:** Predictors of managers' anxiety symptoms in logistic panel regression (*n* = 812, *N* = 406).

	**Anxiety**														
	**(1)**	**(2)**	**(3)**	**(4)**	**(5)**	**(6)**	**(7)**	**(8)**	**(9)**	**(10)**	**(11)**	**(12)**	**(13)**	**(14)**	**(15)**
Gender (reference group: male)	0.69^***^	0.80^***^	0.92^***^	0.81^***^	0.94^***^	0.67^***^	0.67^***^	0.79^***^	0.77^**^	0.69^***^	0.72^***^	0.92^***^	0.94^***^	0.75^***^	0.79^***^
	(0.19)	(0.20)	(0.24)	(0.21)	(0.25)	(0.20)	(0.20)	(0.23)	(0.24)	(0.20)	(0.20)	(0.24)	(0.25)	(0.20)	(0.24)
Age	−0.04^***^	−0.04^***^	−0.05^***^	−0.04^***^	−0.05^***^	−0.05^***^	−0.05^***^	−0.05^***^	−0.05^***^	−0.04^***^	−0.05^***^	−0.05^***^	−0.05^***^	−0.04^***^	−0.05^***^
	(0.01)	(0.01)	(0.01)	(0.01)	(0.01)	(0.01)	(0.01)	(0.01)	(0.01)	(0.01)	(0.01)	(0.01)	(0.01)	(0.01)	(0.01)
Education	−0.15	−0.34	−0.18	−0.41	−0.21	−0.08	−0.09	−0.04	0.00	−0.06	−0.09	−0.05	−0.12	−0.16	−0.06
	(0.33)	(0.34)	(0.38)	(0.35)	(0.40)	(0.34)	(0.34)	(0.37)	(0.40)	(0.34)	(0.35)	(0.38)	(0.40)	(0.34)	(0.39)
Number of children	0.14	0.13	0.16	0.13	0.17	0.14	0.14	0.16	0.17	0.14	0.15	0.18^*^	0.19	0.12	0.15
	(0.08)	(0.08)	(0.09)	(0.08)	(0.10)	(0.08)	(0.08)	(0.09)	(0.10)	(0.08)	(0.08)	(0.09)	(0.10)	(0.08)	(0.09)
Population density	−0.00	−0.00	−0.00	−0.00	−0.00	−0.01	−0.01	−0.01^*^	−0.01	−0.01	−0.01^*^	−0.01	−0.01	0.00	−0.01
	(0.00)	(0.00)	(0.00)	(0.00)	(0.00)	(0.00)	(0.01)	(0.01)	(0.01)	(0.00)	(0.00)	(0.00)	(0.00)	(0.00)	(0.01)
GDP per capita	−0.00	−0.00	−0.00	−0.00	−0.00	−0.00	−0.00	−0.00	−0.00	−0.00	−0.00	−0.00	−0.00	0.00	−0.00^***^
	(0.00)	(0.00)	(0.00)	(0.00)	(0.00)	(0.00)	(0.00)	(0.00)	(0.00)	(0.00)	(0.00)	(0.00)	(0.00)	(0.00)	(0.00)
Cumulative cases (log)		1.17^***^													
		(0.27)													
Cumulative cases per million			0.00^***^												
			(0.00)												
Cumulative deaths (log)				1.43^***^											
				(0.34)											
Cumulative deaths per million					0.02^***^										
					(0.00)										
New cases (log)						−0.65^***^									
						(0.14)									
New cases smoothed (log)							−0.92^***^								
							(0.16)								
New deaths (log)								−0.99^***^							
								(0.15)							
New deaths smoothed (log)									−1.27^***^						
									(0.18)						
New cases per million										−0.04^***^					
										(0.01)					
New cases smoothed per million											−0.07^***^				
											(0.01)				
New deaths per million												−0.64^***^			
												(0.10)			
New deaths smoothed per million													−0.72^***^		
													(0.10)		
Reproduction rate														6.14^***^	
														(0.88)	
Stringency index															−0.18^***^
															(0.02)
Constant	2.03	−5.99	3.46	−2.23	3.13	5.48	7.16^*^	4.69	5.10	2.24	2.70	2.29	2.73	−7.65^**^	23.67^***^
	(2.48)	(3.63)	(2.85)	(3.37)	(2.94)	(2.95)	(3.15)	(3.12)	(3.35)	(2.65)	(2.84)	(2.87)	(3.00)	(2.93)	(4.38)
Country	Yes	Yes	Yes	Yes	Yes	Yes	Yes	Yes	Yes	Yes	Yes	Yes	Yes	Yes	Yes
*N*	812	812	812	812	812	812	812	812	812	812	812	812	812	812	812
Log-likelihood	−431.197	−417.273	−386.643	−414.674	−378.454	−418.275	−409.500	−390.544	−379.905	−415.791	−397.364	−389.424	−374.425	−400.808	−376.647
AIC	912.393	886.546	825.286	881.349	808.908	888.549	870.999	833.089	811.809	883.582	846.728	830.848	800.85	853.616	805.295
BIC	029.881	1008.733	947.473	1003.536	931.095	1010.736	993.186	955.276	933.996	1005.769	968.915	953.035	923.037	975.803	927.482

*Standard errors in parentheses*.

* *p < 0.05*,

** *p < 0.01*,

*** *p < 0.001*.

**Table 4:** Predictors of managers' depression symptoms in logistic panel regression (*n* = 812, *N* = 406).

	**Depression**														
	**(1)**	**(2)**	**(3)**	**(4)**	**(5)**	**(6)**	**(7)**	**(8)**	**(9)**	**(10)**	**(11)**	**(12)**	**(13)**	**(14)**	**(15)**
Gender (reference group: male)	0.37^*^	0.49^**^	0.60^**^	0.53^**^	0.62^**^	0.34	0.36	0.47^*^	0.46^*^	0.36	0.42^*^	0.57^**^	0.60^**^	0.46^*^	0.45^*^
	(0.18)	(0.19)	(0.21)	(0.20)	(0.22)	(0.18)	(0.19)	(0.20)	(0.22)	(0.19)	(0.20)	(0.21)	(0.22)	(0.19)	(0.21)
Age	−0.02^**^	−0.02^**^	−0.03^***^	−0.03^**^	−0.04^***^	−0.03^***^	−0.03^***^	−0.03^***^	−0.04^***^	−0.03^**^	−0.03^***^	−0.03^**^	−0.04^***^	−0.03^**^	−0.03^***^
	(0.01)	(0.01)	(0.01)	(0.01)	(0.01)	(0.01)	(0.01)	(0.01)	(0.01)	(0.01)	(0.01)	(0.01)	(0.01)	(0.01)	(0.01)
Education	−0.07	−0.17	−0.07	−0.28	−0.07	0.03	0.06	0.11	0.12	0.05	0.07	0.14	0.08	−0.04	0.09
	(0.30)	(0.34)	(0.37)	(0.35)	(0.37)	(0.32)	(0.32)	(0.34)	(0.35)	(0.32)	(0.35)	(0.34)	(0.36)	(0.32)	(0.34)
Number of children	0.05	0.04	0.07	0.04	0.08	0.05	0.05	0.06	0.07	0.06	0.07	0.09	0.09	0.04	0.04
	(0.07)	(0.07)	(0.08)	(0.08)	(0.08)	(0.07)	(0.07)	(0.08)	(0.08)	(0.07)	(0.08)	(0.08)	(0.09)	(0.07)	(0.08)
Population density	−0.00	−0.00	0.00	−0.02	0.00	−0.01^*^	−0.01^*^	−0.01^*^	−0.01^*^	−0.01	−0.01^*^	−0.00	−0.00	0.01^*^	−0.01
	(0.00)	(0.01)	(0.00)	(0.01)	(0.00)	(0.00)	(0.01)	(0.00)	(0.01)	(0.00)	(0.00)	(0.00)	(0.00)	(0.00)	(0.01)
GDP per capita	0.00	0.00	−0.00	0.00	0.00	−0.00	−0.00	−0.00	−0.00	0.00	0.00	0.00	0.00	0.00^***^	−0.00^***^
	(0.00)	(0.00)	(0.00)	(0.00)	(0.00)	(0.00)	(0.00)	(0.00)	(0.00)	(0.00)	(0.00)	(0.00)	(0.00)	(0.00)	(0.00)
Cumulative cases (log)		3.61^***^													
		(0.43)													
Cumulative cases per million			0.00^***^												
			(0.00)												
Cumulative deaths (log)				4.83^***^											
				(0.48)											
Cumulative deaths per million					0.04^***^										
					(0.00)										
New cases (log)						−1.10^***^									
						(0.14)									
New cases smoothed (log)							−1.65^***^								
							(0.17)								
New deaths (log)								−1.51^***^							
								(0.13)							
New deaths smoothed (log)									−2.00^***^						
									(0.15)						
New cases per million										−0.05^***^					
										(0.01)					
New cases smoothed per million											−0.11^***^				
											(0.01)				
New deaths per million												−0.97^***^			
												(0.09)			
New deaths smoothed per million													−1.14^***^		
													(0.09)		
Reproduction rate														8.80^***^	
														(0.88)	
Stringency index															−0.27^***^
															(0.02)
Constant	−0.30	−32.06^***^	1.20	−21.54^***^	0.76	5.46	8.95^*^	3.20	5.13	−0.08	0.58	−0.40	−0.02	−14.83^***^	30.20^***^
	(2.28)	(5.01)	(2.57)	(4.53)	(2.51)	(3.21)	(3.76)	(3.39)	(3.78)	(2.59)	(3.03)	(2.52)	(2.58)	(2.99)	(4.00)
Country	Yes	Yes	Yes	Yes	Yes	Yes	Yes	Yes	Yes	Yes	Yes	Yes	Yes	Yes	Yes
*N*	812	812	812	812	812	812	812	812	812	812	812	812	812	812	812
Log-likelihood	−539.654	−477.933	−415.192	−454.928	−395.173	−500.982	−468.122	−431.493	−391.919	−503.032	−448.703	−430.303	−383.499	−469.246	−399.082
AIC	1,129.307	1,007.867	882.385	961.855	842.346	1,053.965	988.243	914.986	835.837	1,058.063	949.407	912.607	818.997	990.491	850.164
BIC	1246.795	1130.054	1004.572	1084.042	964.533	1176.152	1,110.43	1037.173	958.024	1180.25	1071.594	1034.794	941.184	1112.678	972.351

*Standard errors in parentheses*.

* *p < 0.05*,

** *p < 0.01*,

*** *p < 0.001*.

More importantly, Model 2 in both Tables 3, 4 shows that cumulative confirmed cases positively predicted managers' anxiety (b = 5.42; 95% CI: 3.92–6.91; *p* < 0.001) and depression (b = 8.11; 95% CI: 6.81–9.41; *p* < 0.001) symptoms. Model 3 in Tables 3, 4 shows that cumulative confirmed cases per million positively predicted managers' anxiety (b = 0.00; 95% CI: 0.001–0.002; *p* < 0.001) and depression (b = 0.00; 95% CI: 0.002–0.002; *p* < 0.001) symptoms. Similarly, Model 4 in both Tables 3, 4 shows that cumulative deaths positively predicted anxiety (b = 6.42; 95% CI: 4.70–8.15; *p* < 0.001) and depression (b = 9.22; 95% CI: 7.88–10.55; *p* < 0.001) symptoms, and Model 5 in Tables 3, 4 shows that cumulative deaths per million also positively predicted symptoms of these disorders (b = 0.02; 95% CI: 0.02–0.03; *p* < 0.001 and b = 0.04; 95% CI: 0.03–0.04; *p* < 0.001).

Interestingly, Models 6 to 15 in Tables 3, 4 show that new confirmed cases negatively predicted managers' anxiety (b = −0.96; 95% CI: −1.30 to −0.61; *p* < 0.001) and depression (b = −1.52; 95% CI: −1.85 to −1.20; *p* < 0.001) symptoms. Smoothed new confirmed cases negatively predicted managers' anxiety (b = −1.47; 95% CI: −1.88 to −1.06; *p* < 0.001) and depression (b = −2.32; 95% CI: −2.71 to −1.92; *p* < 0.001) symptoms. In addition, daily new deaths negatively predicted the occurrence of anxiety (b = −1.18; 95% CI: −1.51 to −0.84; *p* < 0.001) and depression (b = −1.82; 95% CI: −2.11 to −1.52; *p* < 0.001) symptoms, respectively. Smoothed daily deaths negatively predicted anxiety (b = −1.55; 95% CI: −1.94 to −1.15; *p* < 0.001) and depression (b = −2.45; 95% CI: −2.80 to −2.09; *p* < 0.001) symptoms. We found corroborating evidence using population-adjusted predictors, i.e., new confirmed cases per million, smoothed new cases per million, new deaths per million, and smoothed new deaths per million.

The reproduction rate of COVID-19 positively predicted managers' anxiety (b = 6.20; 95% CI: 4.46–7.94; *p* < 0.001) and depression (b = 9.00; 95% CI: 7.23–10.77; *p* < 0.001) symptoms. Interestingly, the stringency index negatively predicted anxiety (b = −0.18; 95% CI: −0.23 to −0.13; *p* < 0.001) and depression (b = −0.29; 95% CI: −0.34 to −0.24; *p* < 0.001).

Finally, we compared the relative goodness fit of all models that include more predictors than the baseline Model 1 in Tables 3, 4. The cumulative count of deaths emerged as the most suitable predictor of managers' anxiety symptoms since both AIC and BIC for Model 4 were lower than for any alternative model. The cumulative count of deaths was also the most suitable predictor of managers' depression symptoms.

Furthermore, we ran supplemental ordinary least squares models predicting the absolute severity scores of GAD-7 and PHQ-9 (i.e., the sum of the scores across all items of each scale) as a robustness check. The results, which are fully consistent with the findings discussed above, are presented in Tables 5, 6.

**Table 5:** Predictors of managers' anxiety symptoms in ordinary least squares regression (*n* = 812, *N* = 406).

	**Anxiety**														
	**(1)**	**(2)**	**(3)**	**(4)**	**(5)**	**(6)**	**(7)**	**(8)**	**(9)**	**(10)**	**(11)**	**(12)**	**(13)**	**(14)**	**(15)**
Gender (reference group: male)	1.53^***^	1.81^***^	1.63^***^	1.78^***^	1.54^***^	1.33^***^	1.27^***^	1.45^***^	1.35^***^	1.43^***^	1.39^***^	1.53^***^	1.53^***^	1.56^***^	1.43^***^
	(0.41)	(0.40)	(0.37)	(0.41)	(0.37)	(0.39)	(0.38)	(0.37)	(0.37)	(0.39)	(0.37)	(0.37)	(0.36)	(0.38)	(0.37)
Age	−0.07^***^	−0.06^**^	−0.06^***^	−0.06^**^	−0.07^***^	−0.08^***^	−0.08^***^	−0.08^***^	−0.08^***^	−0.07^***^	−0.07^***^	−0.07^***^	−0.07^***^	−0.07^***^	−0.07^***^
	(0.02)	(0.02)	(0.02)	(0.02)	(0.02)	(0.02)	(0.02)	(0.02)	(0.02)	(0.02)	(0.02)	(0.02)	(0.02)	(0.02)	(0.02)
Education	−0.34	−0.78	−0.35	−1.08	−0.42	−0.04	0.02	−0.00	0.14	−0.07	−0.14	−0.27	−0.19	−0.18	−0.10
	(0.69)	(0.67)	(0.61)	(0.68)	(0.61)	(0.65)	(0.63)	(0.63)	(0.62)	(0.66)	(0.62)	(0.62)	(0.61)	(0.63)	(0.62)
Number of children	0.14	0.13	0.15	0.11	0.13	0.15	0.13	0.14	0.15	0.15	0.15	0.16	0.14	0.10	0.09
	(0.16)	(0.15)	(0.14)	(0.16)	(0.14)	(0.15)	(0.15)	(0.14)	(0.14)	(0.15)	(0.14)	(0.14)	(0.14)	(0.15)	(0.14)
Population density	0.00	0.01	0.01	0.01	0.01	−0.01	−0.01	−0.01	−0.01	−0.01	−0.02^*^	−0.00	−0.00	0.02^*^	−0.00
	(0.01)	(0.01)	(0.01)	(0.01)	(0.01)	(0.01)	(0.01)	(0.01)	(0.01)	(0.01)	(0.01)	(0.01)	(0.01)	(0.01)	(0.01)
GDP per capita	0.00	−0.00	−0.00	−0.00	0.00	−0.00	−0.00	−0.00	−0.00	0.00	0.00	0.00	0.00	0.00^***^	−0.00^***^
	(0.00)	(0.00)	(0.00)	(0.00)	(0.00)	(0.00)	(0.00)	(0.00)	(0.00)	(0.00)	(0.00)	(0.00)	(0.00)	(0.00)	(0.00)
Cumulative cases (log)		3.55^***^													
		(0.38)													
Cumulative cases per million			0.00^***^												
			(0.00)												
Cumulative deaths (log)				4.42^***^											
				(0.39)											
Cumulative deaths per million					0.06^***^										
					(0.00)										
New cases (log)						−2.15^***^									
						(0.23)									
New cases smoothed (log)							−2.70^***^								
							(0.23)								
New deaths (log)								−2.25^***^							
								(0.15)							
New deaths smoothed (log)									−2.86^***^						
									(0.15)						
New cases per million										−0.10^***^					
										(0.01)					
New cases smoothed per million											−0.19^***^				
											(0.01)				
New deaths per million												−1.25^***^			
												(0.09)			
New deaths smoothed per million													−1.55^***^		
													(0.08)		
Reproduction rate														16.06^***^	
														(1.27)	
Stringency index															−0.31^***^
															(0.02)
Constant	8.57	−17.63^**^	10.78^*^	−6.32	9.69^*^	18.04^***^	19.77^***^	13.07^**^	13.37^**^	8.46	9.03	9.02	8.91	−18.12^***^	45.85^***^
	(5.26)	(5.82)	(4.68)	(5.36)	(4.67)	(5.09)	(4.93)	(4.79)	(4.75)	(5.03)	(4.70)	(4.77)	(4.67)	(5.24)	(5.22)
Country	Yes	Yes	Yes	Yes	Yes	Yes	Yes	Yes	Yes	Yes	Yes	Yes	Yes	Yes	Yes
*N*	812	812	812	812	812	812	812	812	812	812	812	812	812	812	812
*R^2^*	0.075	0.162	0.303	0.181	0.349	0.169	0.219	0.268	0.334	0.155	0.266	0.261	0.351	0.232	0.308

*Standard errors in parentheses*.

* *p < 0.05*,

** *p < 0.01*,

*** *p < 0.001*.

**Table 6:** Predictors of managers' depression symptoms in ordinary least squares regression (*n* = 812, *N* = 406).

	**Depression**														
	**(1)**	**(2)**	**(3)**	**(4)**	**(5)**	**(6)**	**(7)**	**(8)**	**(9)**	**(10)**	**(11)**	**(12)**	**(13)**	**(14)**	**(15)**
Gender (reference group: male)	1.79^***^	2.13^***^	1.92^***^	2.07^***^	1.80^***^	1.54^***^	1.46^***^	1.69^***^	1.57^***^	1.66^***^	1.61^***^	1.80^***^	1.79^***^	1.84^***^	1.66^***^
	(0.48)	(0.46)	(0.41)	(0.45)	(0.39)	(0.45)	(0.44)	(0.42)	(0.39)	(0.46)	(0.42)	(0.42)	(0.39)	(0.43)	(0.40)
Age	−0.08^***^	−0.06^**^	−0.07^***^	−0.06^**^	−0.07^***^	−0.09^***^	−0.09^***^	−0.09^***^	−0.09^***^	−0.08^***^	−0.08^***^	−0.07^***^	−0.07^***^	−0.07^***^	−0.08^***^
	(0.02)	(0.02)	(0.02)	(0.02)	(0.02)	(0.02)	(0.02)	(0.02)	(0.02)	(0.02)	(0.02)	(0.02)	(0.02)	(0.02)	(0.02)
Education	−0.46	−0.99	−0.47	−1.27	−0.56	−0.08	0.01	−0.02	0.16	−0.11	−0.20	−0.37	−0.27	−0.24	−0.15
	(0.81)	(0.77)	(0.68)	(0.76)	(0.66)	(0.76)	(0.73)	(0.70)	(0.66)	(0.77)	(0.70)	(0.70)	(0.65)	(0.72)	(0.67)
Number of children	−0.01	−0.02	−0.00	−0.05	−0.02	−0.00	−0.02	−0.00	0.01	0.01	0.01	0.02	−0.01	−0.06	−0.07
	(0.19)	(0.18)	(0.16)	(0.17)	(0.15)	(0.18)	(0.17)	(0.16)	(0.15)	(0.18)	(0.16)	(0.16)	(0.15)	(0.17)	(0.16)
Population density	−0.01	0.00	0.00	−0.00	−0.00	−0.03^**^	−0.03^**^	−0.02^**^	−0.03^**^	−0.02^*^	−0.03^***^	−0.01	−0.01	0.01	−0.01
	(0.01)	(0.01)	(0.01)	(0.01)	(0.01)	(0.01)	(0.01)	(0.01)	(0.01)	(0.01)	(0.01)	(0.01)	(0.01)	(0.01)	(0.01)
GDP per capita	0.00	−0.00	−0.00	0.00	0.00	−0.00	−0.00	−0.00	−0.00	0.00	0.00	0.00	0.00	0.00^***^	−0.00^***^
	(0.00)	(0.00)	(0.00)	(0.00)	(0.00)	(0.00)	(0.00)	(0.00)	(0.00)	(0.00)	(0.00)	(0.00)	(0.00)	(0.00)	(0.00)
Cumulative cases (log)		4.28^***^													
		(0.44)													
Cumulative cases per million			0.00^***^												
			(0.00)												
Cumulative deaths (log)				4.90^***^											
				(0.45)											
Cumulative deaths per million					0.07^***^										
					(0.00)										
New cases (log)						−2.73^***^									
						(0.26)									
New cases smoothed (log)							−3.48^***^								
							(0.26)								
New deaths (log)								−2.90^***^							
								(0.17)							
New deaths smoothed (log)									−3.64^***^						
									(0.18)						
New cases per million										−0.13^***^					
										(0.01)					
New cases smoothed per million											−0.24^***^				
											(0.02)				
New deaths per million												−1.59^***^			
												(0.10)			
New deaths smoothed per million													−1.97^***^		
													(0.10)		
Reproduction rate														20.98^***^	
														(1.45)	
Stringency index															−0.40^***^
															(0.02)
Constant	9.44	−22.15^***^	12.24^*^	−7.09	10.84^*^	21.49^***^	23.85^***^	15.25^**^	15.54^**^	9.29	10.03	10.01	9.87^*^	−25.42^***^	57.18^***^
	(6.18)	(6.68)	(5.22)	(5.97)	(5.03)	(5.92)	(5.68)	(5.33)	(5.04)	(5.85)	(5.38)	(5.39)	(4.98)	(6.00)	(5.74)
Country	Yes	Yes	Yes	Yes	Yes	Yes	Yes	Yes	Yes	Yes	Yes	Yes	Yes	Yes	Yes
*N*	812	812	812	812	812	812	812	812	812	812	812	812	812	812	812
*R^2^*	0.061	0.162	0.332	0.182	0.377	0.173	0.236	0.304	0.385	0.160	0.288	0.287	0.392	0.258	0.351

*Standard errors in parentheses*.

* *p < 0.05*,

** *p < 0.01*,

*** *p < 0.001*.

As an additional robustness check, we re-ran all models while including measures for firm size as well as firms' changes in revenues and profits due to COVID-19. None of these additional predictors were significant, and none of the other results changed.

## Discussion

This paper—which is the first to join public country-level COVID-19 statistics with a primary cohort cross-country survey to predict managers' mental disorders across countries—offers several insights that may help to understand manager's mental health across countries to better allocate mental health assistance resources.

First off, consistent with prior studies on other populations (12, 31, 32), gender and age were predictors of anxiety and depression symptoms in managers. For one, female managers were more likely to suffer from mental health problems than male managers. This is in line with the general literature on gender risk for mental health issues, which outlines various risk factors such as social expectations and biological givens that may explain a generally greater vulnerability of females (31–33). This literature also highlights the important role of stressful life events (34), which might of course have become more frequent during a pandemic. Our findings are further consistent with the emerging body of literature specific to the impact of COVID-19, which suggests that females may have a particular underlying vulnerability to negative emotions (24, 35) and are concerned more about economic burdens (35, 36) during the pandemic, compared to their male counterparts. All these factors may of course also promote mental health issues in female managers. For another, younger managers in our sample experienced greater mental distress, potentially because younger managers might have less experience with crisis situations and might thus have developed fewer coping techniques. Again, this finding is compatible with prior literature (12, 37). Our findings thus advise medical professionals to target younger and female managers with mental health service offerings. Notably, other predictors found in the literature, such as education (38) or the number of children (39), failed to predict mental health problems among managers across countries.

More importantly, this study examined country-level COVID-19 severity statistics as predictors of managers' mental health. As mentioned before, managers are a largely neglected vulnerable population that bears responsibility for guiding subordinates, potentially impacting the lives—and the mental health—of many (15). Our findings indicate that cumulative confirmed cases and deaths positively predict anxiety and depression symptoms for those managers during the COVID-19 pandemic, while daily new confirmed cases and deaths negatively predict these mental disorders. Surprisingly, thus, cumulative counts and daily new counts predict managers' mental health in opposing directions. The finding that cumulative counts are positively related with symptoms of mental health issues is fairly intuitive, since a growing cumulative count indicates that the overall magnitude of the COVID-19 crisis as an ongoing historic event increases. Managers might thus be adversely affected by the cumulation of pandemic-related stressors like lockdown measures over time (40). The finding regarding new daily counts is somewhat less intuitive and does not have any precedent in the literature, making it all the more intriguing. The likely most plausible explanation is that managers observing higher daily new counts anticipate satisfactory government intervention, possibly leading to a reduction in concerns over the situation (41). An alternative, but entirely speculative, explanation would be that managers, frequently working remotely during the pandemic, are reminded of their privileged positions by seeing that while daily new counts wreak havoc elsewhere, they themselves and their closer environments have thus far not been affected. This could lead to positive effects on mental health by way of downward comparison with less fortunate workers (42, 43).

Further, the virus reproduction rate positively predicts managers' anxiety and depression, which is intuitive because it directly reflects the speed of spread of COVID-19 and might thus affect the perception of whether the pandemic is controllable. Such control perceptions have repeatedly been linked to mental health consequences (44–46). Again, possibly surprisingly, however, the stringency index negatively predicted managers' mental disorders. In line with our speculation above, this might indicate that measures like school closures, workplace closures, and travel bans can assure people that the crisis is being dealt with and thus decrease managers' concerns about becoming infected or concerns about managing uncertainty in the workplace. This finding is novel compared to previous studies focusing on the general public (47, 48). A possible explanation is that we concentrate on a population with specific skills and views (49–51) that may thus interpret and cope with different indicators differently than the general population.

Finally, we identified cumulative deaths as the most suitable predictor for managers' mental health among the studied variables. Thus, healthcare service providers and human resource departments of multinational companies might particularly wish to use this simple and readily available statistic to prioritize help offerings to managers, at least in the earlier phases of a pandemic. Specifically, multinational companies might want to offer personal protective equipment, online consultation including cognitive behavioral therapy, or telemedicine services to their managers or provide them with other wellness resources to manage stress and improve coping, including workshops and self-help groups to reduce workplace-related stressors (12, 52). Such measures could provide managers with effective coping techniques like problem-focused coping (e.g., planning on what to change about the situation), self-supported emotional coping (e.g., learning to live with the situation), and social-support emotional coping (e.g., getting emotional support from others) (53).

## Limitations and Future Research

There are several limitations to this study. First, we only collected two waves of data, restricting our ability to make causal claims. Although it is a cohort study, future scholars may track individuals' mental health over more waves with shorter intervals. Second, respondents were alumni of one of the most selective consulting firms in the world. It also is skewed heavily toward Western Europe and the United States. Hence, our sample is likely not representative of the overall global population of managers. Others might thus wish to replicate our findings in different manager populations to ensure generalizability. Third, our survey was voluntary, so the response rate was limited, and it is possible that managers with severe mental illness might not have responded in the first place. Additionally, we also did not collect data on respondents' prior psychiatric or psychological treatments, as well as chronic diseases that might affect their risk when contracting COVID-19. The generalizability of our findings might thus be restricted, and future researchers might wish to account for such information. Fourth, we collected only limited data on the organizations the managers were working in. This implies that future researchers might fruitfully replicate our research while, for example, accounting explicitly for organizations' specific responses to the pandemic including any organizational support managers might have received. Fifth, this study aims to explore epidemic statistics as predictors of mental health, and as the first study to do so, we did not extensively explore the possible mechanisms leading to mental health disorders. Our findings thus call for future research to further examine the nature of the relationship between epidemic statistics and mental health.

## Conclusion

In conclusion, this study identified readily available country-level pandemic statistics as predictors of managers' mental health disorder symptoms. Specifically, cumulative COVID-19 statistics predict symptoms positively, while non-cumulative daily statistics predict the same symptoms negatively. The reproduction rate and the stringency index in each country also predicted mental health. These identified country-level predictors can be instrumental for managers' superiors, multinational firms' human resource departments, mental health organizations, and policymakers. In particular, the predictors are readily available from public sources and can help optimize resource allocation and mobilization across geographies during the COVID-19 pandemic.

## Data Availability Statement

The original contributions presented in the study are included in the article/supplementary materials, further inquiries can be directed to the corresponding author/s.

## Ethics Statement

The study was approved (#2020-0401 and #2020-06-01) at ESCP Business School. The patients/participants provided their written informed consent to participate in this study.

## Author Contributions

LG-V collected the survey data. LL ran the analysis. All authors had access to the data and a role in writing the manuscript, designed the study, drafted the manuscript, and reviewed, edited, and approved the final article.

## Funding

We acknowledge financial support by Deutsche Forschungs-gemeinschaft and TU Dortmund University within the funding program Open Access Costs.

## Conflict of Interest

The authors declare that the research was conducted in the absence of any commercial or financial relationships that could be construed as a potential conflict of interest. The reviewer Y-JW declared a shared affiliation with one of the authors, LG-V, to the handling editor at time of review.

## Publisher's Note

All claims expressed in this article are solely those of the authors and do not necessarily represent those of their affiliated organizations, or those of the publisher, the editors and the reviewers. Any product that may be evaluated in this article, or claim that may be made by its manufacturer, is not guaranteed or endorsed by the publisher.

## References

[1] Dai H Zhang SX Looi KH Su R Li J. Perception of health conditions and test availability as predictors of adults' mental health during the covid-19 pandemic: a survey study of adults in Malaysia. Int J Environ Res Public Health. (2020) 17:5498. 10.3390/ijerph17155498 32751459 PMC7432791

[2] Jahanshahi AA Dinani MM Madavani AN Li J Zhang SX. The distress of Iranian adults during the Covid-19 pandemic – More distressed than the Chinese and with different predictors. Brain Behav Immun. (2020) 87:124–5. 10.1016/j.bbi.2020.04.081 32360603 PMC7189859

[3] Gierc M Riazi NA Fagan MJ Di Sebastiano KM Kandola M Priebe CS. Strange days: adult physical activity and mental health in the first two months of the COVID-19 pandemic. Front Public Health. (2021) 9:325. 10.3389/fpubh.2021.567552 33937160 PMC8082023

[4] Gramaglia C Marangon D Azzolina D Guerriero C Lorenzini L Probo M. The mental health impact of 2019-nCOVID on healthcare workers from north-eastern Piedmont, Italy. Focus on burnout Front Public Health. (2021) 9:483. 10.3389/fpubh.2021.667379 34046391 PMC8144493

[5] Lateef T Chen J Tahir M Lateef TA Chen BZ Li J. Typhoon eye effect versus ripple effect: the role of family size on mental health during the COVID-19 pandemic in Pakistan. Global Health. (2021) 17:1–8. 10.1186/s12992-021-00685-5 33781286 PMC8006139

[6] Zhang SX Chen J Jahanshahi AA Alvarez-Risco A Dai H Li J. Succumbing to the COVID-19 pandemic: healthcare workers not satisfied and intend to leave their jobs. Int J Ment Health Addiction. (2021). 10.1007/s11469-020-00418-6 33437225 PMC7790354

[7] Guido CA Amedeo I Avenoso F Bruni J Zicari AM Loffredo L. Risk factors and mental health promotion strategies in children during COVID-19. Front Public Health. (2020) 8:573. 10.3389/fpubh.2020.580720 33178664 PMC7593512

[8] Yáñez JA Afshar Jahanshahi A Alvarez-Risco A Li J Zhang SX. Anxiety, distress, and turnover intention of healthcare workers in Peru by their distance to the epicenter during the COVID-19 crisis. Am J Trop Med Hyg. (2020) 103:1614–20. 10.4269/ajtmh.20-0800 32815512 PMC7543861

[9] Gong H Zhang SX Nawaser K Jahanshahi AA Xu X Li J. The mental health of healthcare staff working during the COVID-19 crisis: their working hours as a boundary condition. J Multidiscip Healthc. (2021) 14:1073. 10.2147/JMDH.S297503 34007182 PMC8122682

[10] Zhang SX Liu J Jahanshahi AA Nawaser K Yousefi A Li J. At the height of the storm: Healthcare staff's health conditions and job satisfaction and their associated predictors during the epidemic peak of COVID-19. Brain Behav Immun. (2020) 87:144–6. 10.1016/j.bbi.2020.05.010 32387345 PMC7199703

[11] Zhao Y Guo J Liu S Aizezi M Zeng Q Sidike A. Prevalence and related factors of depression, anxiety, acute stress, and insomnia symptoms among medical staffs experiencing the second wave of COVID-19 pandemic in Xinjiang, China. Front Public Health. (2021) 9:489. 10.3389/fpubh.2021.671400 34079787 PMC8165275

[12] Han Z Tang X Li X Shen Y Li L Wang J. COVID-19-related stressors and mental health among Chinese college students: a moderated mediation model. Front Public Health. (2021) 9:803. 10.3389/fpubh.2021.586062 34222162 PMC8253361

[13] Yan J Kim S Zhang SX Foo MD Alvarez-Risco A Del-Aguila-Arcentales S. Hospitality workers' COVID-19 risk perception and depression: a contingent model based on transactional theory of stress model. Int J Hosp Manage. (2021) 95:102935. 10.1016/j.ijhm.2021.102935 PMC9756832 36540684

[14] Sun S Zhang SX Jahanshahi AA Jahanshahi M. Drilling under the COVID−19 pandemic: a diary study of professional football players' mental health and workout performance. Stress Health. (2021) 38:3–18. 10.1002/smi.3059 33945206 PMC8237014

[15] Graf-Vlachy L Sun S Zhang SX. Predictors of managers' mental health during the COVID-19 pandemic. Eur J Psychotraumatol. (2020) 11:1834195. 10.1080/20008198.2020.1834195 33244365 PMC7682649

[16] Ivancevich JM Matteson MT Preston C. Occupational stress, type a behavior, and physical well being. Acad Manage J. (1982) 25:373–91. 10.5465/255998 10255815

[17] Egede LE Ruggiero KJ Frueh BC. Ensuring mental health access for vulnerable populations in COVID era. J Psychiatr Res. (2020) 129:147–8. 10.1016/j.jpsychires.2020.07.011 32912595 PMC7360513

[18] Palmer S. Managers More Likely to Be Diagnosed With a Mental Health Condition Than Other Employees. People Management (2019). Available online at: https://www.peoplemanagement.co.uk/news/articles/managers-more-likely-diagnosed-mental-health-condition-other-employees (accessed November 7, 2020).

[19] Ke Y-T Hung C-H. Factors that affect the health status of health care providers—a cross-sectional design. J Nurs Manag. (2020) 28:1199–206. 10.1111/jonm.13057 32473069

[20] Chen J Farah N Dong RK Chen RZ Xu W Yin J. Mental health during the COVID-19 crisis in africa: a systematic review and meta-analysis. Int J Environ Res Public Health. (2021) 18:10604. 10.3390/ijerph182010604 34682357 PMC8536091

[21] Pappa S Chen J Barnett J Chang A Dong RK Xu W. A systematic review and meta-analysis of the mental health symptoms during the Covid-19 pandemic in Southeast Asia. Psychiatry Clin Neurosci. (2021) 76:41–50. 10.1101/2021.06.03.21258001 34704305 PMC8661667

[22] Zhang SX Chen J. Scientific evidence on mental health in key regions under the COVID-19 pandemic–meta-analytical evidence from Africa, Asia, China, Eastern Europe, Latin America, South Asia, Southeast Asia, and Spain. Eur J Psychotraumatol. (2021) 12:2001192. 10.1080/20008198.2021.2001192 34900123 PMC8654399

[23] Zhang SXO Miller SO Xu W Yin A Chen B Delios A. Meta-analytic evidence of depression and anxiety in Eastern Europe during the COVID-19 pandemic. Eur J Psychotraumatol. (2021). 10.1101/2021.06.21.21259227 35186214 PMC8856103

[24] Daly M Sutin AR Robinson E. Longitudinal changes in mental health and the COVID-19 pandemic: evidence from the UK household longitudinal study. Psychol Med. (2020) 1–10. 10.1017/S0033291720004432 33183370 PMC7737138

[25] Spitzer RL Kroenke K Williams JBW Löwe B. A brief measure for assessing generalized anxiety disorder: the GAD-7. Arch Intern Med. (2006) 166:1092. 10.1001/archinte.166.10.1092 16717171

[26] Kroenke K Spitzer RL Williams JBW. The PHQ-9. J Gen Intern Med. (2001) 16:606–13. 10.1046/j.1525-1497.2001.016009606.x 11556941 PMC1495268

[27] Kroenke K Spitzer RL Williams JBW Löwe B. The patient health questionnaire somatic, anxiety, and depressive symptom scales: a systematic review. Gen Hosp Psychiatry. (2010) 32:345–59. 10.1016/j.genhosppsych.2010.03.006 20633738

[28] Chen J Zhang SX Wang Y Jahanshahi AA Dinani MM Madavani AN. The relationship between age and mental health among adults in Iran during the COVID-19 pandemic. Int J Ment Health Addict. (2021) 22:1–16. 10.1007/s11469-021-00571-6 34177394 PMC8218786

[29] Roser M Ritchie H Ortiz-Ospina E Hasell J. Coronavirus Pandemic (COVID-19). Our World in Data (2020). Available online at: https://ourworldindata.org/coronavirus. Available online at: https://ourworldindata.org/coronavirus (accessed December 19, 2020).

[30] Hsiao C. Analysis of Panel Data. 3rd ed. Cambridge: Cambridge University Press (2014). Available online at: https://www.cambridge.org/core/books/analysis-of-panel-data/A774C63FF969DA1944A3F91501702C65 (accessed November 7, 2020).

[31] Kinrys G Wygant LE. Anxiety disorders in women: does gender matter to treatment? Braz J Psychiatry. (2005) 27:s43–50. 10.1590/S1516-44462005000600003 16302053

[32] Remes O Brayne C Linde R van der Lafortune L. A systematic review of reviews on the prevalence of anxiety disorders in adult populations. Brain Behav. (2016) 6:e00497. 10.1002/brb3.497 27458547 PMC4951626

[33] Seeman MV. Mental illness in women. In: Goldman MB, Hatch MC, editors. Women and Health. San Diego, CA: Academic Press (2000). p. 989–96.

[34] Kendler KS Kessler RC Neale MC Heath AC Eaves LJ. The prediction of major depression in women: toward an integrated etiologic model. Am J Psychiatry. (1993) 150:1139. 10.1176/ajp.150.8.1139 8328557

[35] Xiong J Lipsitz O Nasri F Lui LMW Gill H Phan L. Impact of COVID-19 pandemic on mental health in the general population: a systematic review. J Affect Disord. (2020) 277:55–64. 10.1016/j.jad.2020.08.001 32799105 PMC7413844

[36] Almeida M Shrestha AD Stojanac D Miller LJ. The impact of the COVID-19 pandemic on women's mental health. Arch Womens Ment Health. (2020) 23:741–8. 10.1007/s00737-020-01092-2 33263142 PMC7707813

[37] Shechory Bitton M Laufer A. Mental health and coping in the shadow of the COVID-19 pandemic: the Israeli case. Front Public Health. (2021) 8:1016. 10.3389/fpubh.2020.568016 33511096 PMC7835659

[38] Zhang SX Wang Y Rauch A Wei F. Unprecedented disruption of lives and work: health, distress and life satisfaction of working adults in China one month into the COVID-19 outbreak. Psychiatry Res. (2020) 288:112958. 10.1016/j.psychres.2020.112958 32283450 PMC7146665

[39] Ahmad A Rahman I Agarwal M. Early psychosocial predictors of mental health among Indians during coronavirus disease 2019 outbreak. J Health Sci. (2020) 10:147–56. 10.17532/jhsci.2020.950

[40] Pierce M Hope H Ford T Hatch S Hotopf M John A. Mental health before and during the COVID-19 pandemic: a longitudinal probability sample survey of the UK population. Lancet Psychiatry. (2020) 7:883–92. 10.1016/S2215-0366(20)30308-4 32707037 PMC7373389

[41] Mækelæ MJ Reggev N Dutra N Tamayo RM Silva-Sobrinho RA Klevjer K. Perceived efficacy of COVID-19 restrictions, reactions and their impact on mental health during the early phase of the outbreak in six countries. R Soc Open Sci. (2020) 7:200644. 10.1098/rsos.200644 32968525 PMC7481706

[42] Wills TA. Downward comparison as a coping mechanism. In: Snyder CR, Ford CE, editors. Coping With Negative Life Events: Clinical and Social Psychological Perspectives the Plenum Series on Stress and Coping. Boston, MA: Springer US (1987). p. 243–68.

[43] VanderZee KI Buunk BP Sanderman R. Social comparison as a mediator between health problems and subjective health evaluations. Br J Soc Psychol. (1995) 34:53–65. 10.1111/j.2044-8309.1995.tb01048.x 7735732

[44] Southwick FS Southwick SM. The loss of a sense of control as a major contributor to physician burnout: a neuropsychiatric pathway to prevention and recovery. JAMA Psychiatry. (2018) 75:665–6. 10.1001/jamapsychiatry.2018.0566 29799941

[45] Hovenkamp-Hermelink JHM Jeronimus BF van der Veen DC Spinhoven P Penninx BWJH Schoevers RA. Differential associations of locus of control with anxiety, depression and life-events: a five-wave, nine-year study to test stability and change. J Affect Disord. (2019) 253:26–34. 10.1016/j.jad.2019.04.005 31009845

[46] Wierenga KL Moore SE Pressler SJ Hacker ED Perkins SM. Associations between COVID-19 perceptions, anxiety, and depressive symptoms among adults living in the United States. Nurs Outlook. (2021) 69:755–66. 10.1016/j.outlook.2021.03.020 33894985 PMC8530452

[47] O'Hara L Rahim HFA Shi Z. Gender and trust in government modify the association between mental health and stringency of social distancing related public health measures to reduce COVID-19: a global online survey. medRxiv. (2020). 10.1101/2020.07.16.20155200

[48] Ssentongo P Fronterre C Geronimo A Greybush SJ Mbabazi PK Muvawala J. Tracking and predicting the African COVID-19 pandemic. medRxiv. (2020). 10.1101/2020.11.13.20231241 33236036 PMC7685354

[49] Carter JR Irons MD. Are economists different, and if so, why? J Econ Perspect. (1991) 5:171–7. 10.1257/jep.5.2.171

[50] Johnson JEV Powell PL. Decision making, risk and gender: are managers different? Br J Manag. (1994) 5:123–38. 10.1111/j.1467-8551.1994.tb00073.x

[51] Kaplan SN Sorensen M. Are CEOs different? Characteristics of top managers. Natl Bureau Econ Res. (2017). 10.3386/w2383233114445

[52] Pfefferbaum B North CS. Mental health and the COVID-19 pandemic. N Engl J Med. (2020) 383:510–2. 10.1056/NEJMp2008017 32283003

[53] Zheng Y-B Shi L Lu Z-A Que J-Y Yuan K Huang X-L. Mental health status of late-middle-aged adults in China during the coronavirus disease 2019 pandemic. Front Public Health. (2021) 9:628. 10.3389/fpubh.2021.643988 34123986 PMC8187778

